# IL-1β neutralization prevents diastolic dysfunction development, but lacks hepatoprotective effect in an aged mouse model of NASH

**DOI:** 10.1038/s41598-022-26896-3

**Published:** 2023-01-07

**Authors:** Dániel Kucsera, Viktória E. Tóth, Nabil V. Sayour, Tamás Kovács, Tamás G. Gergely, Mihály Ruppert, Tamás Radovits, Alexandra Fábián, Attila Kovács, Béla Merkely, Péter Ferdinandy, Zoltán V. Varga

**Affiliations:** 1 Department of Pharmacology and Pharmacotherapy, Semmelweis University, Budapest, Hungary; 2 HCEMM-SE Cardiometabolic Immunology Research Group, Semmelweis University, Budapest, Hungary; 3 MTA-SE Momentum Cardio-Oncology and Cardioimmunology Research Group, Semmelweis University, Budapest, Hungary; 4 Heart and Vascular Center, Semmelweis University, Budapest, Hungary; 5 Pharmahungary Group, Szeged, Hungary

**Keywords:** Pharmacology, Non-alcoholic steatohepatitis, Interleukins

## Abstract

Interleukin-1β (IL-1β) is a key mediator of non-alcoholic steatohepatitis (NASH), a chronic liver disease, and of systemic inflammation-driven aging. IL-1β contributes to cardio-metabolic decline, and may promote hepatic oncogenic transformation. Therefore, IL-1β is a potential therapeutic target in these pathologies. We aimed to investigate the hepatic and cardiac effects of an IL-1β targeting monoclonal antibody in an aged mouse model of NASH. 24 months old male C57Bl/6J mice were fed with control or choline deficient (CDAA) diet and were treated with isotype control or anti-IL-1β Mab for 8 weeks. Cardiac functions were assessed by conventional—and 2D speckle tracking echocardiography. Liver samples were analyzed by immunohistochemistry and qRT-PCR. Echocardiography revealed improved cardiac diastolic function in anti-IL-1β treated mice with NASH. Marked hepatic fibrosis developed in CDAA-fed group, but IL-1β inhibition affected fibrosis only at transcriptomic level. Hepatic inflammation was not affected by the IL-1β inhibitor. PCNA staining revealed intensive hepatocyte proliferation in CDAA-fed animals, which was not influenced by neutralization of IL-1β. IL-1β inhibition increased hepatic expression of *Pd-1* and *Ctla4*, while *Pd-l1* expression increased in NASH. In conclusion, IL-1β inhibition improved cardiac diastolic function, but did not ameliorate features of NASH; moreover, even promoted hepatic immune checkpoint expression, with concomitant NASH-related hepatocellular proliferation.

## Introduction

Non-alcoholic fatty liver disease (NAFLD) has become the most common chronic liver disease^1^. Initially, it is characterized by hepatosteatosis, which is benign and reversible; however, 25% of NAFLD cases progresses into steatohepatitis (NASH)^2^. During NASH, complex pro-inflammatory and pro-fibrotic events occur, which may deteriorate into cirrhosis^3^, and even into hepatocellular carcinoma (HCC). Most patients with NASH have a higher risk for cardiovascular mortality, due to the concomitant presence of CV risk factors^4^. Additionally, age-related systemic, chronic, low-grade inflammation – so called inflamm-aging – is a major contributor to both chronic liver and cardiac diseases, including NASH and heart failure^5–8^. Interleukin-1β (IL-1β) is a key mediator of both inflamm-aging and cardiometabolic inflammation, during which its serum concentration is low and is maintained for a prolonged time. However, upon liver injury, both parenchymal and non-parenchymal cells are able to release high amount of IL-1β^9,10^. Thus both systemic circulation-derived and the locally released IL-1β contributes with several mechanisms to the pathophysiology of NASH, such as promotion of steatosis^11^, interference with insulin signaling^12^, stimulation of hepatic stellate cells to produce fibrotic proteins^13^, and by promotion of neutrophil recruitment^14^.

Beside NASH, IL-1β has been recognized as a key factor in cardiovascular diseases, such as atherosclerosis^15^, acute myocardial infarction^16^ and heart failure as well^17,18^. In the landmark CANTOS trial, it has been proven that cardiovascular mortality can be reduced by attenuating IL-1β-driven inflammation^19^. Heart failure with preserved ejection fraction (HFpEF) is driven by metabolic co-morbidities (similarly to NASH) and by systemic inflammation^20^. Diastolic dysfunction is a key functional feature of HFpEF, to which systemic inflammation-derived IL-1β may contribute significantly^21^, implicating the potential therapeutic use of IL-1β blockade in this disease as well.

Early recognition of the importance of IL-1β in NASH led to several investigations, assessing the inflammasome-caspase-interleukin-1β pathway, as a potential therapeutic target^10,22–24^. Despite extensive experimental and clinical studies, NASH is still lacking effective treatment options^25^. Furthermore, treatment of hepatocellular carcinoma, a severe consequence of NASH, is still not entirely resolved, as it is a type of cancer that is resistant to immunotherapies^26,27^.

In light with the cardinal role of IL-1β in both NASH^28^ and age-related cardiometabolic diseases^29^, we aimed to investigate the effects of anti-IL-1β monoclonal antibody treatment on cardiac function and the potential disease altering effect in an aged non-obese mouse model of NASH, that included the evaluation of hepatic microenvironment upon IL-1β blockade.

## Results

### Anti-IL-1β treatment improves cardiac diastolic function

NASH is closely linked to insulin resistance, hypertension, atherogenic dyslipidemia and obesity, all of which are established cardiovascular risk factors, and are key drivers in the development of diastolic dysfunction. However, the individual contribution of each of these components to cardiac dysfunction is not known. It is important to highlight that cardiovascular events are the main cause of mortality in patients with NASH and NAFLD^30^. To assess whether cardiac dysfunction occurs in the current model of NASH in aging mice, and to evaluate the cardiac effects of IL-1β blockade in this disease, here we performed conventional echocardiography followed by strain analyses with 2D speckle tracking echocardiography in a non-obese mouse model of NASH.

The most often used dietary models of NASH are the methionine and choline deficient (MCD), the choline deficient L-amino acid defined (CDAA) and the high fat diet (HFD) models^31^. MCD induces steatohepatitis with marked fibrosis in 3–5 weeks; however, leads to severe weight loss. HFD necessitates 20 weeks of feeding and develops only steatosis with minimal fibrosis and leads to significant weight gain. CDAA diet is neutral in terms of weight change; however, characteristic steatohepatitis and fibrosis develops within a relatively short period of feeding (8–10 weeks)^32^. In our study, we aimed to study the effect of NASH without the metabolic and pro-inflammatory burden of obesity and insulin resistance, thus we have chosen the CDAA-induced model of NASH.

In our model, systolic functions remained unchanged in the CDAA diet group compared to CON diet group, and were unaffected by anti-IL-1β treatment, as indicated by the preservation of cardiac output, ejection fraction, GLS and GCS.

**Figure 1:**
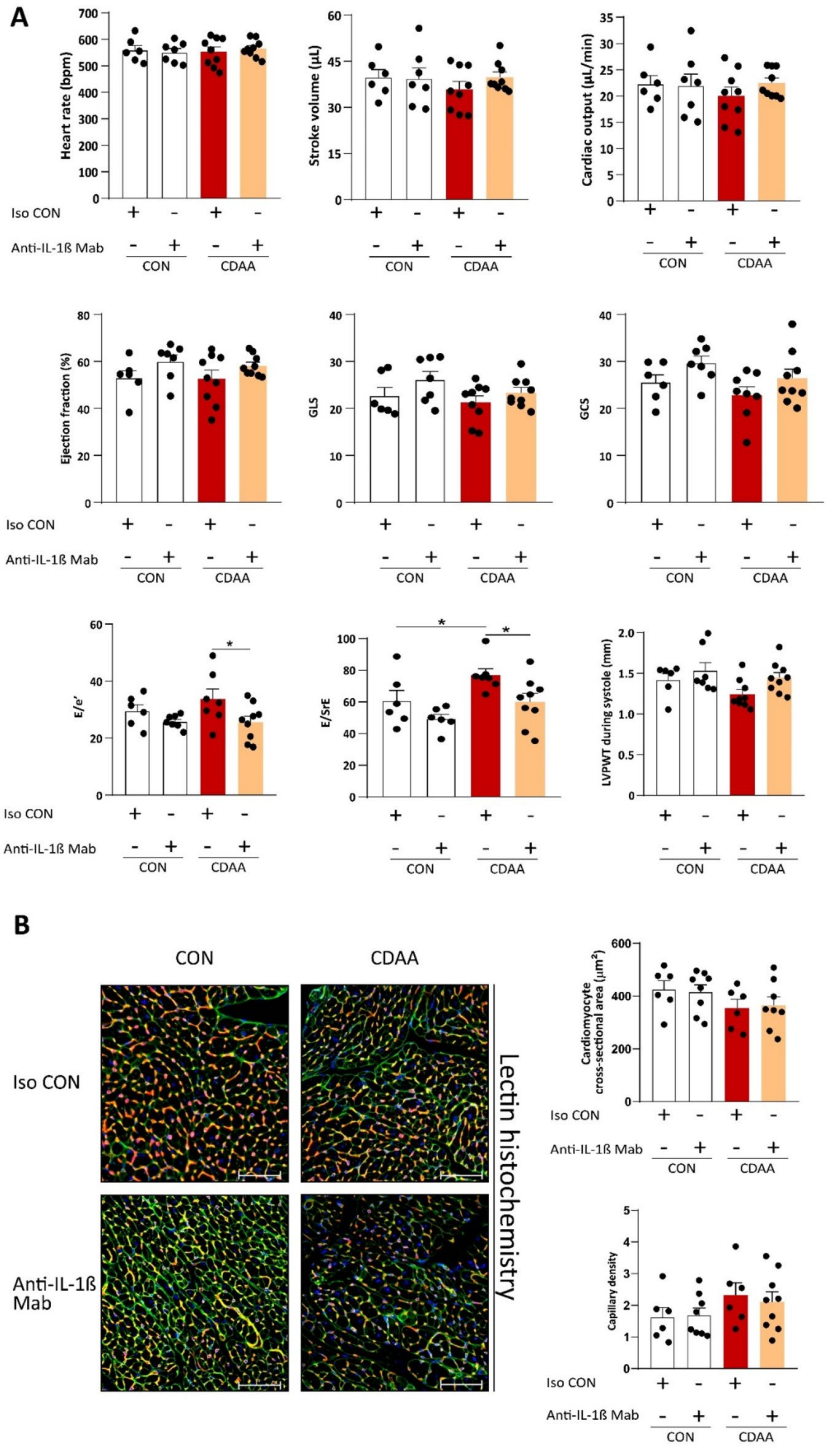
Cardiac function of mice in a CDAA diet-induced NASH model. Cardiac function assessment with conventional echocardiography and strain analysis with 2D speckle tracking (**A**) (n = 6–9/group). Cardiac lectin histochemistry. Green shows cardiac cell membrane, orange shows cardiac capillary endothelial cells, blue shows nuclei (**B**) (n = 6–9/group). CDAA, Choline Deficient L-Amino Acid-defined diet; CON, control diet; Iso CON, isotype control; Anti-IL-1β, anti-interleukin-1β monoclonal antibody; GLS, global longitudinal strain; GCS, global circumferential strain; SrE, early diastolic strain rate; LVPWT, left ventricular posterior wall thickness. Two-way ANOVA, Fisher’s LSD post hoc test, *P < 0.05, **P < 0.01, ***P < 0.001.

E/e’ ratio, an indirect parameter of LV filling pressure, did not change by the CDAA diet. The early mitral inflow velocity-to-early diastolic strain rate (E/SrE) a marker previously shown to be superior to E/e’ in humans with aortic stenosis for the assessment of diastolic dysfunction^33^, was markedly increased in the CDAA diet group compared to the CON diet group, indicating diastolic dysfunction, which was reversed by anti-IL-1β treatment (Fig. 1A).

Cardiac remodeling is a paramount driver of functional and structural myocardial deterioration. To assess structural changes in the heart we performed cardiac lectin histochemistry. We measured the cross-sectional area (CSA) of cardiomyocytes by wheat germ agglutinin staining (a marker of cell membrane), then we assessed the capillary density (CD) by isolectin B4 staining (a marker of cardiac endothelial cells). Neither CSA nor CD showed difference between the groups (Fig. 1B).

In summary, inhibition of IL-1β improved diastolic function in aged animals with NASH, while systolic function was unaffected by both NASH and IL-1β blockade. Complete results of the echocardiographic measurements are shown in Supplementary Table 1. Additionally, we did not observed cardiac hypertrophy in our animals.

### CDAA diet induced marked hepatic fibrosis, but IL-1β blockade did not affect overall fibrosis

**Figure 2:**
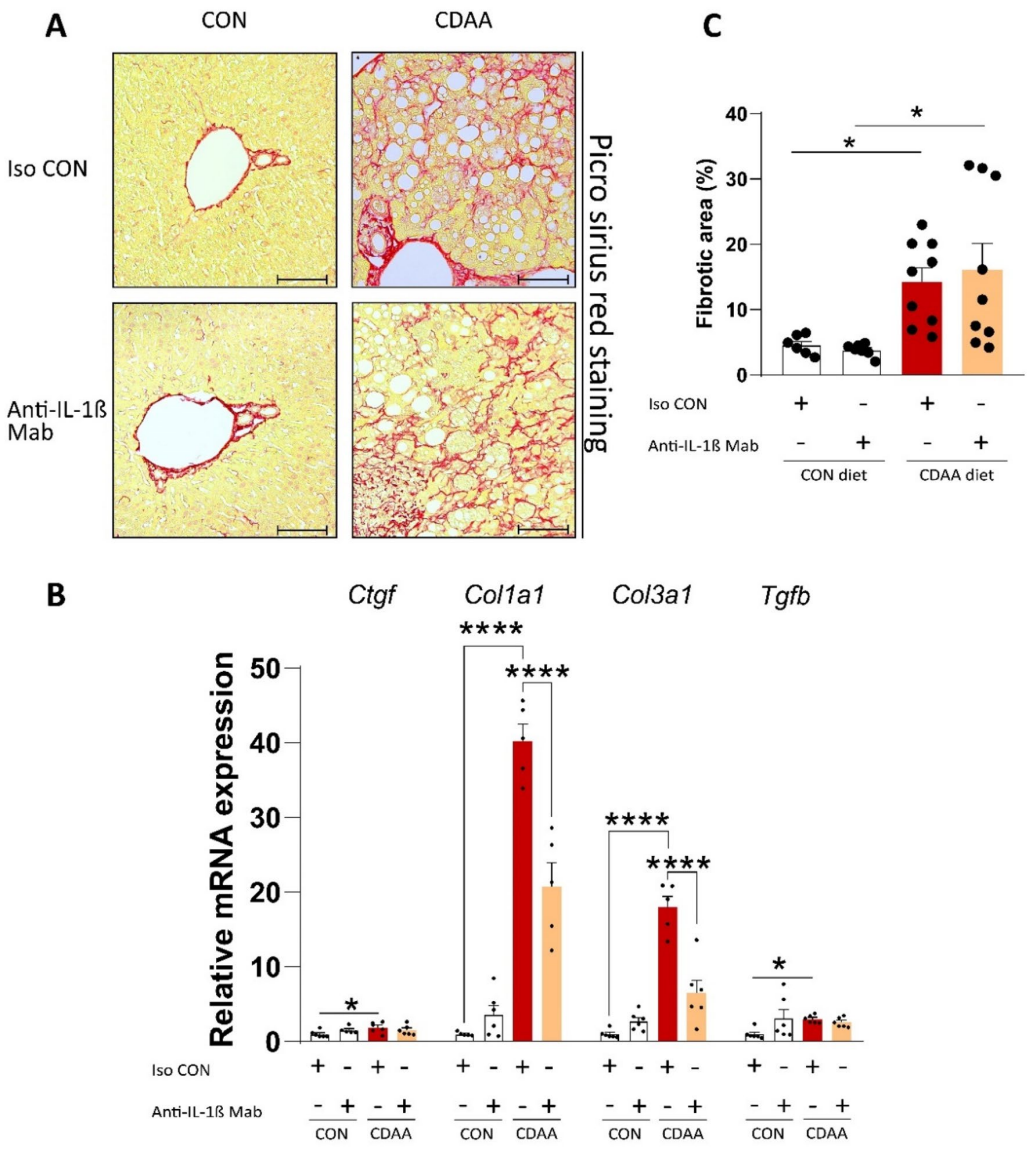
Histological and molecular assessment of hepatic fibrosis. Evaluation of hepatic fibrosis by picrosirius-red staining (**A**) (n = 6–9/group). Analysis of major pro-fibrotic genes, normalized to *Ppia*, with qRT-PCR (**B**) (n = 5–6/group). Quantification of overall fibrosis (**C**) (n = 6–9/group). CDAA, Choline Deficient L-Amino Acid-defined diet; CON, control diet; Iso CON, isotype control; Anti-IL-1β, anti-interleukin-1β monoclonal antibody. Scale bar shows 100 µm. Two-way ANOVA, Fisher’s LSD post hoc test, *P < 0.05, **P < 0.01, ***P < 0.001.

Fibrosis is the most important predictive factor for long-term outcomes of NASH, such as hepatocellular carcinoma and overall mortality^3^. CDAA diet promoted periportal and centrilobular fibrosis (Fig. 2A). Gene expression analysis of key fibrotic markers confirmed the previous finding: expression of connective tissue growth factor (*Ctgf*), type I and type III collagen (*Col1a1*, *Col3a1*, respectively) and transforming growth factor β (*Tgfb*) were elevated in mice with CDAA diet-induced NASH. Anti-IL-1β Mab administration reduced the transcription of both *Col1a1* and *Col3a1*, while both *Ctgf* and *Tgfb* were unaffected by the treatment (Fig. 2B). Quantitative analysis of picrosirius-red staining; however, showed no difference in overall fibrosis due to IL-1β inhibition (Fig. 2C).

In summary, IL-1β blockade decreased the transcription of key fibrotic markers in aged animals, but failed to ameliorate the overall fibrosis.

### Animals fed with CDAA diet develop key features of NASH, which is not affected by IL-1β inhibition

Choline is an important nutrient that participates in packaging and secretion of VLDL particles. In addition, choline is essential in mitochondrial β-oxidation. Therefore, choline deficiency results in aberrant hepatic VLDL secretion and in decreased fatty acid oxidation, both of which contribute to hepatocellular lipid accumulation and, subsequently, leads to steatohepatitis^34^ without inducing metabolic alterations such as obesity and insulin resistance, thus enables us to investigate the sole effects of NASH.

**Figure 3:**
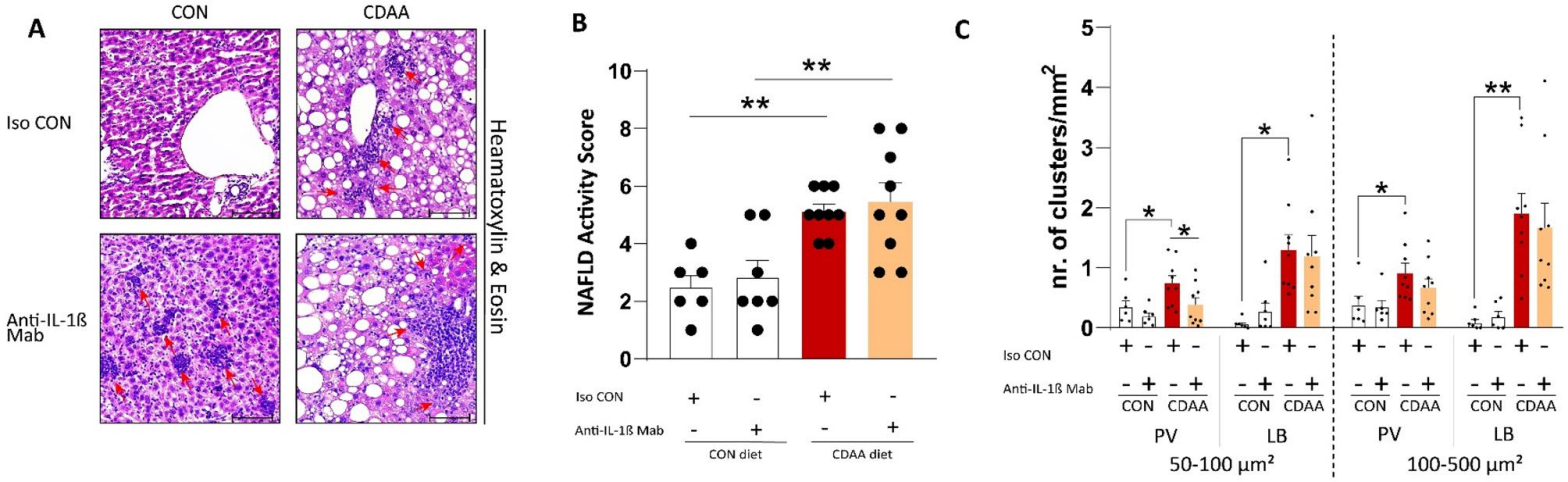
Histological assessment of inflammatory cell infiltrations. Investigation of hepatic inflammatory foci (red arrows) on hematoxylin–eosin stained sections (**A**). NAFLD Activity Score (NAS) (**B**). Evaluation of number, area and anatomical site of the immune cell infiltrations (**C**) (n = 6–9/group). CDAA, Choline Deficient L-Amino Acid-defined diet; CON, control diet; Iso CON, isotype control; Anti-IL-1β, anti-interleukin-1β monoclonal antibody; PV, periportal; LB, lobular. Scale bar shows 100 µm. Two-way ANOVA, Fisher’s LSD post hoc test, *P < 0.05, **P < 0.01, ***P < 0.001.

8 weeks of CDAA diet provoked extensive steatosis and immune cell infiltration into the liver parenchyma (Fig. 3A), and hepatomegaly (Supplementary Table 2). The NAFLD Activity Score (NAS) was not affected by IL-1β blockade (Fig. 3B).

The immune cell clusters were evaluated by their anatomical site, cluster area and number. Aged animals from the CDAA diet fed cohort are characterized by extensive lobular inflammatory cell infiltrations with both small (with an area of 50–100 µm^2^) and large clusters (with an area of 100–500 µm^2^). Pharmacological inhibition of IL-1β did not influence the size and number of the large infiltrations. Marked periportal inflammatory foci were also observed in animals with steatohepatitis. At this anatomical site, IL-1β blockade limited the formation of small immune clusters, while notable difference of large immune infiltration formation was not detected (Fig. 3C).

In conclusion, anti-IL-1β monoclonal antibody treatment could not improve the NAFLD Activity Score and could not induce resolution of already formed large inflammatory foci. However, it may limit the infiltration of additional immune cells, thus preventing the formation of new immune clusters or the growth of already formed clusters.

### Immune clusters are comprised of macrophages and neutrophils

After morphological and anatomical characterization of inflammatory foci, we moved on to further investigate the type of infiltrating immune cells.

**Figure 4:**
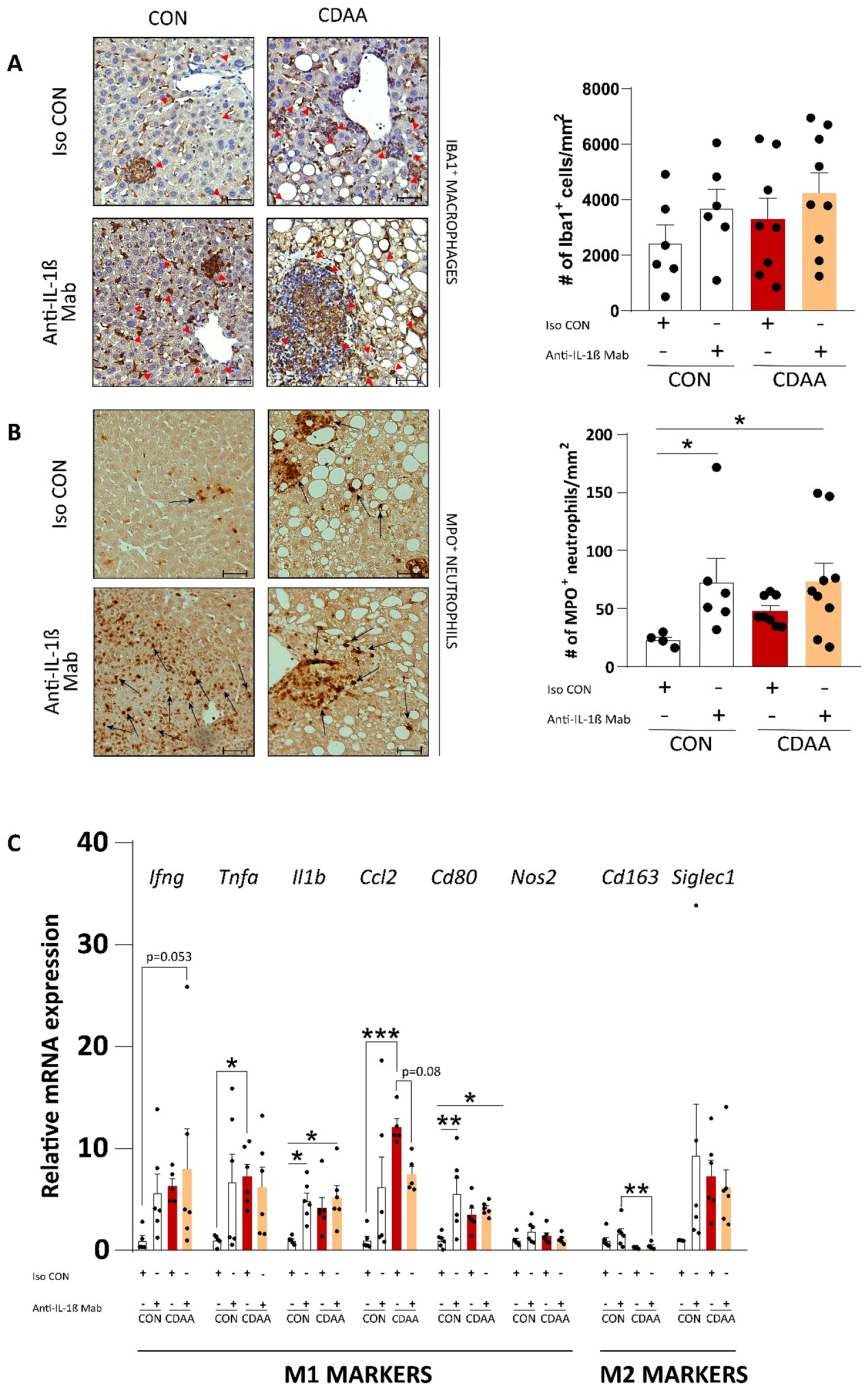
Hepatic macrophage and neutrophil infiltration. Immunohistochemical assessment and quantification of hepatic Iba1^+^ macrophages (**A**) (n = 6–9/group) and MPO^+^ neutrophils (**B**) (n = 4–9/group). Red and black arrows show the corresponding immune cells. qRT-PCR assessment of major M1 and M2 markers (**C**) (n = 5–6/group). CDAA, Choline Deficient L-Amino Acid-defined diet; CON, control diet; Iso CON, isotype control; Anti-IL-1β, anti-interleukin-1β monoclonal antibody, Iba1; ionized calcium-binding adapter molecule 1; MPO, myeloperoxidase. Scale bar shows 100 µm. Two-way ANOVA, Fisher’s LSD post hoc test, *P < 0.05, **P < 0.01, ***P < 0.001.

Iba1 staining revealed no difference between the groups; however, an increasing trend was observed in anti-IL-1β treated aged mice with steatohepatitis. Macrophages participated in hepatic crown-like structure formation and comprised the majority of immune cells within the inflammatory clusters (Fig. 4A). Activated Kupffer cells secrete pro-inflammatory cytokines and chemokines, which promote neutrophil accumulation into the liver^35,36^ and may further contribute to hepatic damage^37^. One of the pro-inflammatory cytokines that initiate neutrophil recruitment is IL-1β, thus we assumed that IL-1β blockade will prevent hepatic neutrophil accumulation. Interestingly, we observed the opposite: in aged mice treated with anti-IL-1β Mab more neutrophils were observed than isotype control treated aged animals (Fig. 4B).

Following histological analyses, we performed gene expression analyses of major markers of pro- and anti-inflammatory phenotypes of macrophages to determine their role in our aged mouse model of NASH and to evaluate how their expression alter upon IL-1β inhibition. Interestingly, IL-1β blockade caused elevation in expression of genes of *Il1b* and *Cd80* (Fig. 4C). The expression pattern of *Il1b* suggests a compensatory increase due to decreased IL-1β signaling. CD80 is a co-stimulatory molecule that is expressed by antigen-presenting cells and initiates activation of both CD8^+^ and FOXP3^+^ T cells. Thus, indirectly, IL-1β blockade plays a dual role: it may contribute to further hepatic damage by increasing cytotoxic activity, or it may dampen inflammation by establishing a regulatory microenvironment^38^.

CDAA fed mice had up-regulated hepatic expression of *Ccl2* gene, which was previously shown being a central chemokine driving hepatic macrophage recruitment^39^. Neutralization of IL-1β caused a trend-like decrease in *Ccl2* expression (Fig. 4C). Gene expression of interferon-γ (encoded by *Ifng*) in aged mice with NASH revealed a tendency of increase upon IL-1β inhibition (Fig. 4C). M1 macrophages express inducible nitric oxide synthase (encoded by *Nos2*), which converts L-arginine into nitric oxide and peroxynitrite, thus contributing to hepatic nitrosative stress^40^. In our study, we did not observe, however, any substantial alteration in the expression level of *Nos2*.

Both CD163 and Siglec1 (also known as CD169) are markers of M2 macrophages, in our study, only *Cd163* was downregulated in mice with NASH, regardless of anti-IL-1β treatment (Fig. 4C).

In summary, we did not observe substantial difference in macrophage numbers between our cohorts. On the other hand, groups treated with IL-1β blocker showed extensive MPO^+^ neutrophil infiltration.

### Both NASH and IL-1β blockade creates a microenvironment that may promote malignant alterations

We continued our investigation by analyzing markers of proliferation, and invasiveness, as well as checked the expression of key immune checkpoints. Inflammation is a fundamental driver of fibrosis, apoptosis and steatohepatitis, all of which may contribute to compensatory hepatic proliferation^41^. Thus we aimed to assess whether hepatic proliferation was present in our model, and if so, to examine the effects of anti-IL-1β Mab treatment on hepatocellular proliferation.

**Figure 5:**
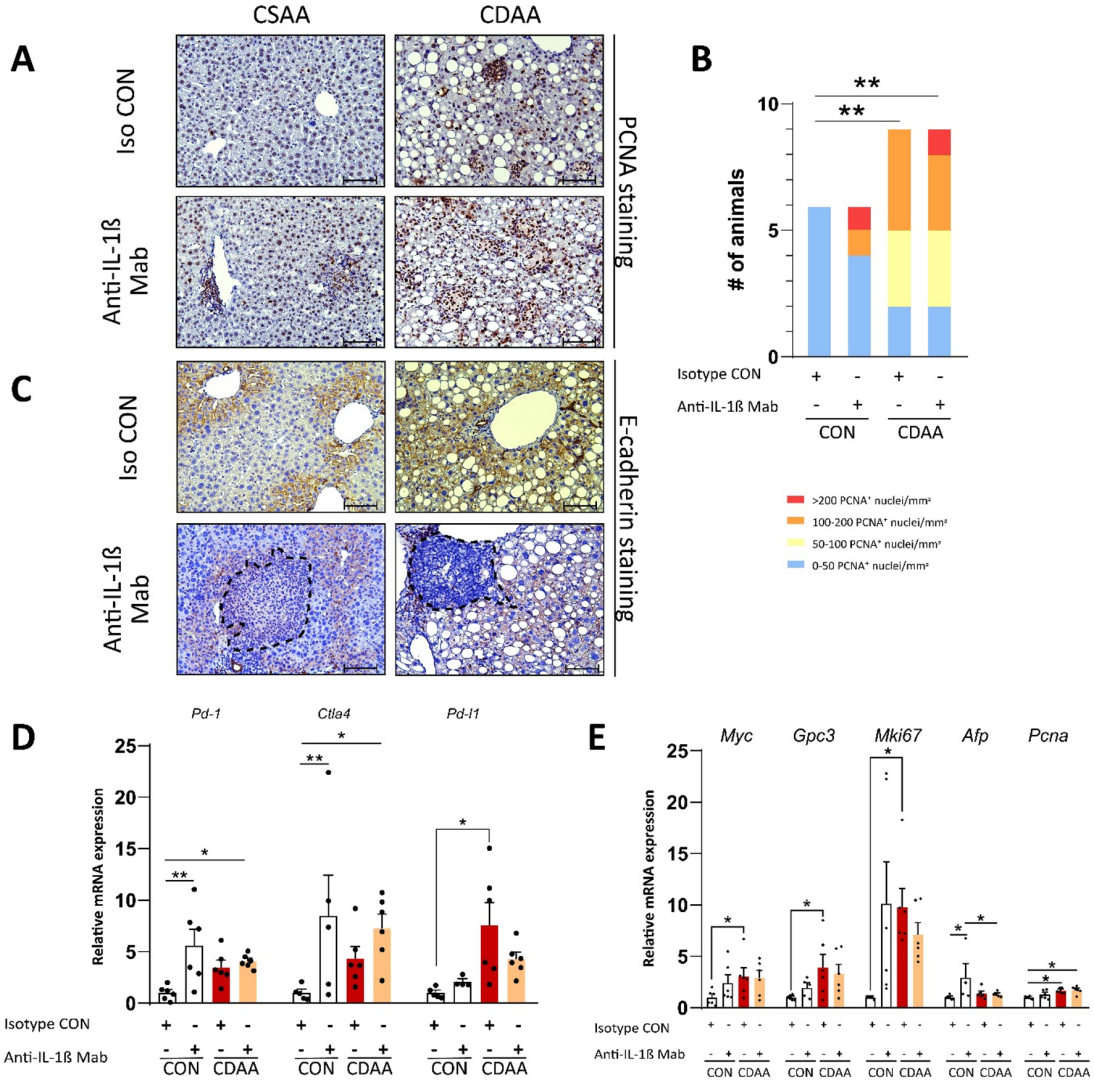
Hepatic microenvironment. Immunohistochemical evaluation of proliferation by assessing PCNA^+^ nuclei (**A**, **B**) and invasiveness by assessing E-cadherin staining (**C**) (n = 6–9/group). qRT-PCR analysis of key immune checkpoints (**D**) and proto-oncogenes (**E**) (n = 4–6/group). CDAA, Choline Deficient L-Amino Acid-defined diet; CON, control diet; Iso CON, isotype control; Anti-IL-1β, anti-interleukin-1β monoclonal antibody; PCNA, proliferating cell nuclear antigen. Scale bar shows 100 µm. Two-way ANOVA, Fisher’s LSD post hoc test, Fisher’s exact test, *P < 0.05, **P < 0.01, ***P < 0.001.

We performed PCNA immunostaining, which unveiled marked hepatocellular proliferation in aged animals fed with CDAA diet (Fig. 5A). Quantification of PCNA^+^ nuclei showed that more animals with more than 100 PCNA^+^ nuclei/mm^2^ were found within the CDAA fed groups (Fig. 5B).

Epithelial-mesenchymal transition (EMT) is a hallmark of tumor malignancy and invasiveness. Immunohistochemistry of E-cadherin showed cell membrane-associated staining at periportal regions of aged animals treated with isotype control; however, in anti-IL-1β Mab treated animals, hepatocytes were found to lose their membrane-bound positivity at sites of inflammatory lesions (Fig. 5C).

Gene expression of pharmacologically targetable immune checkpoints revealed that *Pd-1* and *Ctla4* were up-regulated upon IL-1β blockade; whereas, *Pd-l1* expression was up-regulated only due to the CDAA diet (Fig. 5D).

We continued our transcriptomic analysis by investigating the expression levels of five major markers of proliferation: MYC (*Myc*), Glypican-3 (*Gpc3*), MKi-67 (*Mki67*), α-fetoprotein (*Afp*) and Proliferating Cell Nuclear Antigen (*Pcna*). *Pcna* expression was elevated in aged mice with NASH, thus confirming the previous results of immunohistochemistry (Fig. 5A, 5E). *Myc* and *Mki67* were increased in CDAA-fed control animals, while expression level of *Gpc3* was enhanced in CDAA fed animals in both treatment groups. Interestingly, transcription of *Afp* was enhanced by IL-1β inhibition (Fig. 5E).

Overall, these results suggest that both NASH and anti-IL-1β monoclonal treatment promotes hepatocellular proliferation, epithelial-mesenchymal transition and contributes to the development to a microenvironment that enables immune evasion.

## Discussion

Non-alcoholic fatty liver disease has become the most common liver disease worldwide^42^. Following prolonged hepatocellular stress and damage, liver cells may undergo apoptosis and/or necrosis. The subsequent formation of cellular debris activates Kupffer cells, hepatic stellate cells, thus triggering inflammation and fibrosis. This stage is known as steatohepatitis. During NASH, Kupffer cells initiate the secretion of various pro-inflammatory cytokines and chemokines, including IL-1β. IL-1β is paramount for appropriate inflammatory response during both sterile and non-sterile inflammation. Nonetheless, age-associated systemic inflammation-derived and/or hepatocellular apoptosis-induced prolonged and excessive IL-1β signaling concomitantly contribute to altered hepatic and cardiac physiologic functions. Thus making IL-1β a potential therapeutic target for both diseases^11–14,28,43^.

In our study, we observed an increase in E/SrE ratio in CDAA-fed cohort compared to CON animals. Recently, a similar observation was reported in a mouse model of HFpEF, supporting that strain rate analysis is a sensitive method to measure subtle myocardial functional changes^44^. We report here an improvement of diastolic dysfunction by IL-1β inhibition, observed with speckle tracking echocardiography. This finding is in line with previous studies, where it was shown that cardiac IL-1β signaling can interfere with the transduction pathway of β-adrenergic receptors and may modulate the expression of phospholamban^21,45,46^ resulting in impaired cardiac function.

In contrast to our observations in the heart, we found that IL-1β blockade failed to ameliorate key features of NASH. First of all, we detected, in our experiments, marked fibrosis upon CDAA diet. Hepatic fibrosis is mainly driven by hepatic stellate cells^47^, which produce collagen upon cytokine activation (including IL-1β)^13^ and hepatocellular debris^48^. Additionally, stellate cells secrete various pro-inflammatory cytokines and chemokines, resulting in a vicious cycle of inflammation and fibrosis^47^. Interestingly, IL-1β blockade decreased the transcription of these genes in our model; however, the overall fibrosis remained unaffected. This may suggest an induction of pro-resolution processes on the molecular level by IL-1β inhibition, that does not appear yet macroscopically. During hepatic repair, fibrosis may decrease due to both hepatic stellate cell apoptosis and degradation of fibrotic proteins, resulting in reduction of extracellular matrix deposition (ECM) and degradation of ECM^49^. Previously, it was reported that upstream blockade of IL-1β maturation by NLRP3 inflammasome and caspase-1 inhibition reduced hepatic fibrosis, proving that lacking IL-1β in NASH may ameliorate pro-fibrotic events^50–52^. It is possible, however, that 8 weeks of treatment may not be sufficient to meaningfully reverse fibrosis in our model.

Second of all, our investigation of inflammatory foci suggests that IL-1β neutralization may limit the progression of small immune clusters into large ones. Interestingly, we observed a tendency of increase in macrophage numbers in mice with NASH treated with IL-1β blocker, while *Ccl2* gene expression was slightly diminished by the treatment. The downstream transcription factor of IL-1β, NF-κB, regulates the secretion of CCL2 (also known as MCP-1). In an atherogenic diet-fed NASH model, inhibition of NLRP3 significantly reduced the gene expression of *Ccl2*^51^. Furthermore, the expression of interferon-γ also showed a tendency towards increase in CDAA fed mice treated with the IL-1β blocking antibody. In line with our findings, Hart K. et al. recognized that high levels of IFN-γ, a potent polarizing factor for M1 macrophages and a fundamental driver for Th1 commitment, can be protective against NASH^53^. Surprisingly, IL-1β blockade increased the number of neutrophils, the second major constituent of immune clusters, that is in contrast to previous observations, where mice with NASH treated with NLRP3 inhibitors showed decreased hepatic neutrophil infiltration^51^. A possible explanation for this contradiction might be the direct inhibition of inflammasomes and/or caspase-1 decreases the maturation of both IL-1β and IL-18. Therefore, the unaffected IL-18, in our study, might activate neutrophils as previously reported^54^.

Third of all, we report no change in hepatocyte proliferation upon IL-1β inhibition. The primary role of compensatory proliferation is regeneration of the hepatic architecture to restore lost hepatocytes due to apoptosis^55^. Hepatocellular apoptosis occurs more frequently in patients with NASH and it is a key driver of disease progression^56,57^. Excessive hepatocellular cell death is a known inducer of compensatory hepatocyte proliferation, and subsequent malignant transformation^58^. Accordingly, we observed marked proliferation in aged animals fed with CDAA diet. Pyroptosis, an inflammatory cell death type, can occur upon caspase-1 activation resulting the maturation of IL-1β, which may further promote pyroptosis by positive-feedback. Therefore, IL-1β may support a vicious cycle of inflammatory cell death during NASH. Although caspase-1 inhibitors have been deemed clinically ineffective in NASH^59^, we assumed that if we are able to halt pyroptosis, by interfering with the vicious cycle of IL-1β, we may prevent and/or decrease hepatocellular proliferation. We found here however, that anti-IL-1β treatment does not have considerable effect on hepatocyte proliferation. It is known that NASH is a pivotal etiological factor in the development of hepatocellular carcinoma^26^, a radio- and chemotherapy-resistant tumor^60^. Over the past decade, immunotherapies emerged as potential treatments. Immune checkpoint inhibitors (ICIs) are approved in advanced HCC^61^; however, the immunologic microenvironment of HCC is paramount in achieving effectiveness. ICIs were proven effective in viral HCC, but they lack efficacy in NASH-induced HCC^27^. Currently, this is a basis of intense investigations, to find therapeutic tools that may promote ICI efficiency in NASH-related HCC^62^.

Therefore, we tested whether IL-1β blockade can affect the expression of major immune checkpoints and thus modulate the tissue microenvironment. We detected that NASH increased the expression of *Pd-l1*. This finding coincides with a previous study where it was shown that IL-1β might induce PD-L1 expression on malignant liver cells^63^. Prior studies noted that increased IL-1β level may possess a crucial immunosuppressive role in different tumors, thus its inhibition might prove to be beneficial^19,64–66^. However, in our experiments, IL-1β blockade increased the expression of *Ctla4* and *Pd-1*, suggesting development of an immunosuppressive microenvironment. This is in line with previous clinical observations, where patients with HCC showed poor prognosis when hepatic pro-inflammatory cytokine expression, including IL-1β, was suppressed^67^.

In our study, a clear M1 polarization is visible in animals with NASH (*Il1b* and *Cd80* upregulation), while *Cd163* is down-regulated, which is regarded as pro-tumourigenic M2 macrophage marker. Generally, M2 macrophages are considered pro-tumourigenic; in contrast, Siglec1^+^ cells were shown to have anti-tumourigenic effect against hepatic malignant cells^68,69^. In summary, at cellular level an anti-tumourigenic niche is observed, while at molecular level we see a more immunosuppressive microenvironment.

The literature indicates that IL-1β contributes significantly to cancer-promoting inflammation in a wide variaty of cancers^70^. However, IL-1β also shows anti-tumourigenic effects^70^, thus there is a possibility that IL-1β inhibition might adversely affect cancer development. Th9 cells activated by IL-1β were shown to be highly effective against melanoma cells^71^. Therefore, we might argue that interruption of IL-1β signaling in patients with melanoma might negatively impact the disease progression. IL-R1 deficiency on neutrophils promoted colorectal carcinoma (CRC) progression, consequently, the lack of IL-1β signaling on neutrophils due to neutralization of circulating IL-1β might contribute as well to the progression of CRC^65^. Additionally, it was shown that nasopharyngeal carcinoma cells-derived IL-1β mediates anti-tumour effects on tumour-associated neutrophils, thus IL-1β blockade might prevent this beneficial effect of neutrophils^72^. In a triple-negative breast cancer model IL-1β decreased the viability of breast cancer cell, further supporting that systemic IL-1β inhibition might contribute to cancer progression^73^.

It is clear that IL-1β has a wide range of effects in different cancers and cardiometabolic diseases as well, thus future treatments with IL-1β blockers should take into consideration the aformentioned adverse possibilities.

## Conclusions

Here we report for the first time an improvement in cardiac diastolic function by IL-1β blockade in an aged mouse model of NASH, a disease of paramount burden. Although IL-1β inhibition showed anti-fibrotic effects in the liver at the transcriptional level, it failed to ameliorate other key features of NASH, such as inflammation and increased hepatocyte proliferation. Furthermore, IL-1β blockade deleteriously altered the expression of key immune checkpoints, i.e., led to an increased expression of *Ctla4*, *Pd-1*, creating a milieu for pre-malignant transformation. Therefore, neutralization of IL-1β might not be a beneficial therapeutic target in NASH, and likely does not affect the therapeutic effectiveness of immune checkpoint inhibitors in NASH-induced malignancies.

## Limitation

CDAA diet, although widely used, does not induces key clinical features of NASH, such as hyperglycemia, hypertriglyceridemia, and obesity. However, as we stated before, we purposefully selected the CDAA diet model, because of its lack of obesitogenic potential, thus allowing us to exclude the effects of obesity on cardiac and liver pathologies. Baseline echocardiography and histology were not performed, which would have allowed us to observe the cardiac effects of anti-IL-1β treatment in a time-dependent manner.

## Materials and methods

### Experimental animals, diets and treatments and ethical approval

Young (8 weeks old) C57Bl/6J mice were purchased from the Oncological Research Center, Department of Experimental Pharmacology, Budapest, Hungary. Mice were maintained under 12–12 light–dark cycle under controlled environment (20–24°C and 35–75% relative humidity) in individually ventilated cages, holding 2–4 mice per cage. Standard chow diet and tap water were available ad libitum.

Control (CON) diet (E 15668–04) and Choline Deficient L-Amino Acid defined (CDAA) diet (E15666–94) was purchased from SSNIFF GmbH (Soest, Germany). In short, CDAA diet is composed of crystalline amino acids with no choline and low methionine, and 1% cholesterol content. The energy intake is comprised by 31 kJ% of fats, 58 kJ% of carbohydrates and 11 kJ% of proteins.

Anti-IL-1β monoclonal antibody (BE0246) and the corresponding isotype control (BE0091) were purchased from BioXCell, USA.

All experimental procedures were done in accordance with the Guide for Care and Use of Laboratory Animals published by US National Institutes of Health (NIH publication No. 85–23, revised 1996), with the EU Directive (2010/63/EU), and in compliance with the ARRIVE guidelines, and was approved by the National Scientific Ethical Committee on Animal Experimentation (PE/EA/1912–7/2017, Budapest, Hungary).

### Non-alcoholic steatohepatitis model

**Figure 6:**
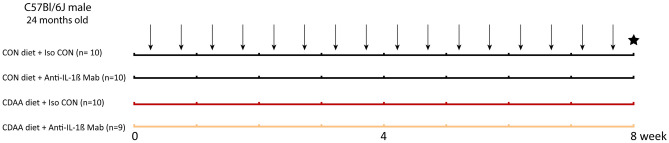
Experimental protocol. 24-months-old C57Bl/6J mice were fed with CDAA or CON diet for 8 weeks. Black arrows indicate intraperitoneal treatments with isotype control or anti-IL-1β Mab (50 µg/mice). The black star indicates the termination of mice, when blood and organ samples were collected. CDAA, Choline Deficient L-Amino Acid-defined diet; CON, control diet; Iso CON, isotype control; Anti-IL-1β, anti-interleukin-1β monoclonal antibody.

Thirty-nine male C57Bl/6J mice were aged up to 24 months of age. One day before study initiation, body weights were measured (weighing on average 37 g). Mice were randomized by body weight and assigned to CON diet-fed group (n = 10) or CDAA diet fed group (n = 10) and were treated with anti-IL-1β Mab (n = 9) or isotype control (n = 10) for 8 weeks. Older male mice were used, due to their susceptibility to frailty and chronic inflammation-driven cardiac function decline^74^. The animals were treated two times per week with a dose of 50 µg/mouse^75^. Prior of termination, echocardiographic evaluation was done to assess cardiac function. Upon sacrifice, internal organs and blood were collected for histological and molecular analyses (Fig. 6).

Regarding mortality, we report that 1 animal died 1 day before the initiation of the study. Additionally, in total 8 mice succumbed (out of which 5 within the first week) during the study. The cause of death is likely due to the advanced age of the animals and/or the stress of handling (intraperitoneal injection). Thus, in total 31 mice remained for echocardiographic, histologic and molecular analyses.

### Echocardiography

Mice were anesthetized with isoflurane (5% for induction, 2% for maintenance), cardiac functions were assessed with the Vevo 3100 high-resolution in vivo imaging system (Fujifilm VisualSonics, Toronto, Canada) with a MX400 transducer. Two-dimensional recordings of the heart were obtained by conventional long-axis view for volumetric analyses, and short-axis views for ventricular diameter and wall thickness analyses. Apical four-chamber view was used to measure diastolic parameters. Early mitral inflow velocity (E) and mitral annular early diastolic velocity (e’) was measured with pulse-wave Doppler and tissue Doppler, respectively.

Stroke volume (SV) was calculated as LVEDV – LVESV. Ejection fraction was assessed as [(LVEDV – LVESV)/LVEDV × 100]. Cardiac output was determined as SV × HR/1000. Left ventricular internal diameter during systole and diastole (LVIDs, LVIDd), anterior and posterior wall thickness (LVAWT, LVPWT) was measured. Fractional shortening was calculated with the following formula: [(LVIDd − LVIDs)/LVIDd] × 100. Left ventricular mass was calculated as {[(LVIDd + AWTd + PWTd)^3^ − LVIDd^3^] × 1.0} × 0.8 + 0.14.

Echocardiographic recordings were evaluated with the VevoLAB software by an operator blinded to the study groups.

### Strain analysis with 2D speckle-tracking

Beyond conventional echocardiographic measurements, two-dimensional (2D) speckle-tracking echocardiography (STE) was performed in order to quantify strain and its temporal derivative strain rate. These parameters enable characterization of different directions of myocardial deformation. High-quality ECG-gated loops of long- and short-axis views of the LV dedicated for speckle tracking were acquired. The recordings were exported to a standalone workstation for offline analysis with commercially available software (2D Cardiac Performance Analysis v1.2, TomTec Imaging Systems, Unterschleissheim, Germany). Speckle-tracking analysis was performed by a single experienced operator blinded to the study subjects’ characteristics.

In order to quantify global longitudinal strain (GLS), three cardiac cycles from the selected long-axis loop were analyzed. To calculate global circumferential strain (GCS), and early diastolic strain rate (SrE), the same measurements were performed using the short-axis recordings. After manual delineation of the endocardial border on the end-diastolic frame, the software automatically divided the region of interest into six segments and tracked them throughout the cardiac cycles. If the operator detected low endocardial tracking fidelity visually, the contour was realigned, and the calculation was repeated to a maximum of three attempts. Systolic longitudinal and circumferential strains, and early diastolic strain rates of the 6 LV segments averaged over the three cardiac cycles were used to calculate the corresponding GLS, GCS and SrE values. E/SrE values were calculated using the E values measured by conventional echocardiography.

### Histologic analysis

Liver and heart samples were fixed in neutral buffered formalin for 24 h, then dehydrated and embedded in paraffin. Five µm thick sections were cut with a microtome and used later on. All staining was visualized and captured with Leica LMD6 microscope (Wetzlar, Germany). In case of liver samples, the specimens’ entire area were scanned and analyzed with 6.3 × magnification, while, in case of heart samples, 5 microphotographs were captured from endocardial regions.

### Hematoxylin and Eosin staining

Paraffin embedded liver sections were deparaffinized, hydrated, and subsequently, stained with hematoxylin and counterstained with eosin. H&E staining was used to assess the area of lipid droplets and inflammatory clusters using ImageJ software. Two liver specimens’ entire area was manually quantified with ImageJ from two different lobes for inflammatory clusters. The area of each cluster was measured. The number of inflammatory clusters from the two lobes was averaged, then categorized according to their anatomical location and area. Scoring of steatosis, hepatocyte ballooning, inflammatory foci and fibrosis was used to determine the NAFLD Activity Score (NAS) as suggested by Kleiner et al.^76^ NAS was obtained as follows: steatosis: < 5%, 5–33%, > 33%, > 66% = score 0–1-2–3; hepatocyte ballooning: none, few, many per 20X microscopic field = score 0–1-2, lobular inflammation: 0, 1–2, 2–4, > 4 per 20X microscopic field = score 0–1-2–3, fibrosis: no fibrosis, mild/moderate pericellular fibrosis or portal/periportal fibrosis, pericellular and portal/periportal fibrosis, bridging fibrosis, cirrhosis = score 0–1-2–3-4.

### Picrosirius-Red staining

Liver sections were stained with 0.0125% picrosirius-red for 1 h, then washed with 1% acetic acid. Quantification of fibrosis was assessed by ImageJ software.

### Immunohistochemistry

Liver sections underwent antigen retrieval (citrate buffer pH = 6 or Tris buffer pH = 9) for 15 min. Endogenous peroxidase was blocked by 3% H_2_O_2_ in PBS. Afterwards, sections were blocked with 2.5% goat or horse serum and 2% milk powder or bovine serum albumin. Primary antibodies – Iba1, marker of macrophages (019–19741, Wako Pure Chemical Industries, Japan); MPO, marker of neutrophils (AF3667, R&D Systems, USA); E-cadherin, marker of epithelial-mesenchymal transition (610181, BD Biosciences, USA); PCNA, marker of proliferation (13110S, Cell Signaling Technology, USA – were diluted (1:2000, 1:200, 1:2000, 1:4000, respectively) in goat or horse serum and were incubated overnight at 4°C. Sections were washed three times with PBS, then the specimens were incubated with the following secondary antibodies: anti-rabbit IgG HRP (8114S, Cell Signaling Technology, USA), anti-goat IgG HRP (MP-7405, Vector Laboratories, USA), anti-mouse HRP (MP-2400, Vector Laboratories, USA), then were washed and signals were developed with diaminobenzidine (ImmPact DAB EqV Peroxidase (HRP) Subrate, Vector Laboratories, Burlingame, CA, United States).

Iba1, MPO and PCNA positivity was quantified with ImageJ software. Recordings were transformed into 16-bit formats, then threshold, particle size and circularity was adjusted to identify individual signals. If signals were clustered, then watersheding function was applied to estimates the border of the individual cells allowing more precise quantification.

### Lectin histochemistry

Lectin histochemistry was performed with wheat germ agglutinin (WGA-FITC – marker of cell membrane, 1:50, Sigma Aldrich, L4895) and with isolectin B4 (ILB4-DyLight 594 – marker of cardiac endothelial cells, 1:50, Invitrogen, L32473). Cardiac antigens were retrieved at pH = 9, then incubated with the aforementioned lectins overnight at 4°C. Five images were captured from the endocardial region per heart. Cardiomyocyte cross-sectional area was automatically segmented with ImageJ Software. Capillary density was calculated as the ratio of capillary count and the average of cardiomyocyte cross-sectional area.

### qRT-PCR

Total RNA isolation was performed with isopropanol/chloroform precipitation method as described earlier^77^. Briefly, cDNA synthesis (Sensifast cDNA synthesis kit, Bioline, London, UK) was carried out from 1 µg of total RNA according to the manufacturer’s instructions. SensiFAST SYBR Green master mix (Bioline, UK) was used to amplify the target genes using a LightCycler® 480 II (Roche, Germany) instrument. Results were obtained by using 2^−ΔΔCp^ evaluation method. Six samples were used per cohort, thus allowing us to analyze 8 genes on a plate of 384 wells. Primer sequences are available in Supplementary Table 3.

### Statistical analysis

All values are presented as mean ± standard error of mean (SEM). Two-way ANOVA followed by Fisher’s LSD post hoc test was used for multiple comparison analyses. Contingency analysis was evaluated by Fisher’s exact test. *P < 0.05 was considered statistically significant. Following each statistical analysis, outliers were identified by ROUT method and were excluded from the analysis. The statistical analyses were performed with GraphPad Prism software.

### Institutional review board statement

All experimental procedures were done in accordance with the Guide for Care and Use of Laboratory Animals published by US National Institutes of Health (NIH publication No. 85-23, revised 1996), with the EU Directive (2010/63/EU), and was approved by the National Scientific Ethical Committee on Animal Experimentation (PE/EA/1912-7/2017, Budapest, Hungary).

## Supplementary Information

Supplementary Table 1.

Supplementary Table 2.

Supplementary Table 3.

## Data Availability

The dataset used and/or analyzed during the current study are available from the corresponding author on reasonable request.
