# Supplementary material for: IL-1β neutralization prevents diastolic dysfunction development, but lacks hepatoprotective effect in an aged mouse model of NASH
Source: Sci Rep. 2023 Jan 7;13:356. doi: 10.1038/s41598-022-26896-3 (PMC9825403; doi:10.1038/s41598-022-26896-3)
Supplement: Supplementary file 1 — Supplementary Table 1. [file 41598_2022_26896_MOESM1_ESM.pdf]

IL-1β neutralization prevents diastolic dysfunction development, but lacks hepatoprotective effect in an aged mouse model of NASH

Dániel Kucsera, PharmD[1,2,3], Viktória E. Tóth, PharmD, PhD[1,2,3], Nabil V. Sayour, MD[1,2,3], Tamás Kovács, MSc [1,2,3], Tamás Gergely, MD [1,2,3], Mihály Ruppert, MD, PhD[4], Tamás Radovits, MD, PhD[4], Alexandra Fábián, MD[4], Attila Kovács, MD, PhD [4], Béla Merkely, MD, PhD[4], Péter Ferdinandy, MD, PhD[1,5], Zoltán V. Varga, MD, PhD[1,2,3]

[1]Department of Pharmacology and Pharmacotherapy, Semmelweis University, Budapest, Hungary;
[2]HCEMM-SE Cardiometabolic Immunology Research Group, Semmelweis University, Budapest, Hungary;
[3]MTA-SE Momentum Cardio-Oncology and Cardioimmunology Research Group, Semmelweis University, Budapest, Hungary;
[4]Heart and Vascular Center, Semmelweis University, Budapest, Hungary;
[5]Pharmahungary Group, Szeged, Hungary.

Corresponding author: Zoltán V. Varga (varga.zoltan@med.semmelweis-univ.hu)

Echocardiography

Group ID		Parasternal long axis B-mode								
		Heart rate (bpm)	Area;s (mm^2)	Area;d (mm^2)	Volume;s (µL)	Volume;d (µL)	Stroke volume (µL)	Ejection fraction (%)	Fractional shortening (%)	Cardiac output (µL/min)
**CON diet + Iso CON**	Mean	559.90	18.11	28.13	37.82	77.63	39.81	53.06	14.77	22.29
	SEM	18.56	2.138	1.98	7.31	9.19	2.64	3.40	2.11	1.68
**CON diet + Anti-IL-1ß mAb**	Mean	551.10	14.49	24.99	25.98	65.45	39.47	60.02	14.00	21.94
	SEM	14.60	0.57	0.75	1.96	3.69	3.46	2.93	1.00	2.32
	P vs. CON diet + Iso CON	*0.7196*	***0.0742***	***0.0983***	***0.0900***	*0.1621*	*0.9303*	*0.1340*	*0,7262*	*0.8925*
**CDAA diet + Iso CON**	Mean	552.70	16.92	25.71	34.33	70.28	35.95	52.64	11.36	19.99
	SEM	17.77	1.41	1.20	5.08	5.67	2.37	3.54	1.35	1.63
	P vs. CON diet + Iso CON	*0.7571*	*0.5266*	*0.1748*	*0.5885*	*0.3678*	*0.3014*	*0.9229*	*0,1081*	*0.3474*
**CDAA diet + Anti-IL-1ß mAb**	Mean	565.20	15.57	25.55	28.92	68.68	39.76	58.18	12.88	22.45
	SEM	11.05	0.72	0.82	2.14	3.44	1.62	1.50	1.19	0.92
	P vs. CON diet + Anti-IL-1β mAb	*0.5255*	*0.5466*	*0.7375*	*0.6340*	*0.6773*	*0.9340*	*0.6556*	*0,5709*	*0.8278*
	P vs. CDAA diet + Iso CON	*0.5477*	*0.4181*	*0.9205*	*0.3514*	*0.8258*	*0.2549*	*0.1584*	*0,4154*	*0.2631*

Group ID		Short axis M-mode												
		Heart rate (bpm)	Diameter;s (mm^2)	Diameter;d (mm^2)	Volume;s (µL)	Volume;d (µL)	Stroke volume (µL)	Ejection fraction (%)	Fractional shortening (%)	LV mass (mg)	LVAW;s (mm)	LVAW;d (mm)	LVPW;s (mm)	LVPW;d (mm)
**CON diet + Iso CON**	Mean	548.50	3.02	4.32	37.49	84.87	47.38	57.34	30.38	185.00	1.45	1.04	1.42	0.97
	SEM	15.17	0.23	0.17	6.74	8.11	3.21	4.56	3.16	18.44	0.08	0.05	0.08	0.06
**CON diet + Anti-IL-1ß mAb**	Mean	540.50	2.46	4.15	22.92	76.86	53.94	71.36	40.97	157.40	1.51	0.98	1.53	0.90
	SEM	16.71	0.19	0.12	4.75	5.09	3.16	4.25	3.20	12.80	0.06	0.02	0.11	0.05
	P vs. CON diet + Iso CON	*0.7322*	***0.0703***	*0.4455*	*0.1014*	*0.4458*	*0.1465*	***0.0241***	***0.0171***	*0.1919*	*0.5512*	*0.4409*	*0.3315*	*0.3497*
**CDAA diet + Iso CON**	Mean	549.80	3.14	4.34	41.39	86.28	44.88	53.99	28.13	173.60	1,50	1.02	1.24	0.90
	SEM	17.97	0.21	0.16	6.65	7.97	2.45	3.73	2,45	13.79	0.08	0.06	0.06	0.03
	P vs. CON diet + Iso CON	*0.9555*	*0.6830*	*0.9156*	*0.6351*	*0.8870*	*0.5524*	*0.5511*	*0.5735*	*0.5632*	*0.6307*	*0.7462*	*0.1215*	*0.3427*
**CDAA diet + Anti-IL-1ß mAb**	Mean	558.10	2.96	4.34	34.74	85.59	50.84	60.04	32.15	185.60	1.54	1.03	1.45	0.99
	SEM	8.41	0.14	0.11	3.61	4.89	2.68	2.93	2.22	9.49	0.05	0.05	0.06	0.05
	P vs. CON diet + Anti-IL-1β mAb	*0.4098*	***0.0794***	*0.3356*	*0.1403*	*0.3602*	*0.4431*	***0.0423***	***0.0272***	*0.1437*	*0.8180*	*0.4907*	*0.4013*	*0.2043*
	P vs. CDAA diet + Iso CON	*0.6749*	*0.4672*	*0.9883*	*0.3689*	*0.9381*	*0.1201*	*0.2339*	*0.2651*	*0.4994*	*0.6800*	*0.8511*	***0.0510***	*0.1890*

Group ID		Short axis B-mode			Diastolic function				Strain analysis with 2D speckle tracking					
		Area;s (mm^2)	Area;d (mm^2)	Fractional Area Change	E' (mm/s)	IVRT (ms)	E (mm/s)	E/E'	Peak GLS	Peak GCS	E/LSrE	E/CSrE	LSrS	CSrS
**CON diet + Iso CON**	Mean	13.06	7.13	46.27	23.09	15.00	662.50	29.58	22.71	25.59	63.67	60.74	8.879	8.969
	SEM	1.29	0.94	2.66	2.41	0.58	49.34	2.24	1.83	1.721	11.97	6.801	0.272	0.796
**CON diet + Anti-IL-1ß mAb**	Mean	12.06	5.82	51.80	24.98	13.90	642.10	25.85	26.13	29.73	44.79	49.34	11.73	11.58
	SEM	0.41	0.40	2.60	1.87	1.03	47.57	0.87	1.812	1.518	6.058	3.005	0.802	0.751
	P vs. CON diet + Iso CON	*0.4016*	*0.2463*	*0.2492*	*0.5945*	*0.4539*	*0,7532*	*0.3031*	*0.146*	*0.139*	*0.121*	*0.149*	***0.029***	***0.052***
**CDAA diet + Iso CON**	Mean	12.67	7.66	40.24	20.12	15.76	635.30	33.71	21.30	22.86	67.45	77.00	7.483	8.889
	SEM	0.70	0.83	3.89	2.51	0.10	41.09	3.49	1.371	1.779	7.662	3.932	0.308	0.794
	P vs. CON diet + Iso CON	*0.7316*	*0.6121*	*0.1860*	*0.4063*	*0.6013*	*0.6749*	*0.2550*	*0.519*	*0.311*	*0.751*	***0.037***	*0.253*	*0.950*
**CDAA diet + Anti-IL-1ß mAb**	Mean	13.54	7.21	47.30	26.94	14,99	654.30	25.62	23.33	26.40	60.66	59.99	11.25	9.979
	SEM	0.64	0.58	2.14	2.38	1.00	35.92	2.11	1.056	1.888	6.323	5.123	1.027	0.912
	P vs. CON diet + Anti-IL-1β mAb	*0.1773*	*0.1740*	*0.2996*	*0.5435*	*0.4151*	*0.8353*	*0.9437*	*0,1871*	*0,1881*	*0,149*	*0.140*	*0.655*	*0.179*
	P vs. CDAA diet + Iso CON	*0.3959*	*0.6353*	***0.0870***	***0.0420***	*0.557*	*0.7458*	***0.0186***	*0,302*	*0,1475*	*0,53*	***0.017***	***0.001***	*0.339*

LSrE = early diastolic strain rate acquired from GLS
CSrE = early diastolic strain rate acquired from GCS
LSrS = systolic strain rate acquired from GLS
CSrS = systolic strain rate acquired from GCS

Supplementary table 1. - Conventional and two-dimensional speckle tracking echocardiography.

Two-dimensional images were recorded in long-axis view for volumetric and longitudinal strain analyses; short-axis view for wall thickness, diameter and circumferential strain analyses. Diastolic parameters were obtained by apical four chamber view. Data is presented as mean ± standard error of mean (SEM). Statistical analysis: two-way ANOVA, Fischer's LSD post hoc test. Statistically significant is considered P < 0.05. Statistically significant P values are highlighted with bold font.
