# Supplementary material for: IL-1β neutralization prevents diastolic dysfunction development, but lacks hepatoprotective effect in an aged mouse model of NASH
Source: Sci Rep. 2023 Jan 7;13:356. doi: 10.1038/s41598-022-26896-3 (PMC9825403; doi:10.1038/s41598-022-26896-3)
Supplement: Supplementary file 2 — Supplementary Table 2. [file 41598_2022_26896_MOESM2_ESM.pdf]

IL-1β neutralization prevents diastolic dysfunction development, but lacks hepatoprotective effect in an aged mouse model of NASH

Dániel Kucsera, PharmD[1,2,3], Viktória E. Tóth, PharmD, PhD[1,2,3], Nabil V. Sayour, MD[1,2,3], Tamás Kovács, MSc [1,2,3], Tamás Gergely, MD [1,2,3], Mihály Ruppert, MD, PhD[4], Tamás Radovits, MD, PhD[4], Alexandra Fábián, MD[4], Attila Kovács, MD, PhD [4], Béla Merkely, MD, PhD[4], Péter Ferdinandy, MD, PhD[1,5], Zoltán V. Varga, MD, PhD[1,2,3]

[1]Department of Pharmacology and Pharmacotherapy, Semmelweis University, Budapest, Hungary;
[2]HCEMM-SE Cardiometabolic Immunology Research Group, Semmelweis University, Budapest, Hungary;
[3]MTA-SE Momentum Cardio-Oncology and Cardioimmunology Research Group, Semmelweis University, Budapest, Hungary;
[4]Heart and Vascular Center, Semmelweis University, Budapest, Hungary;
[5]Pharmahungary Group, Szeged, Hungary.

Corresponding author: Zoltán V. Varga (varga.zoltan@med.semmelweis-univ.hu)

Organ weights

Group ID		Normalized to body weight: Heart weight/body weight (mg/mg)	Liver weight/body weight (mg/mg)	Kidney weight/body weight (mg/mg)	Spleen weight/body weight (mg/mg)
**CON diet + Iso CON**	Mean	0.006267	0.043900	0.006493	0.003687
	SEM	0.0005063	0.0051930	0.0004917	0.0006103
**CON diet + Anti-IL-1ß**	Mean	0.00612	0.057500	0.007380	0.003890
	SEM	0.0002202	0.0089780	0.0002626	0.0006608
P vs. CON diet + Iso CON		*0.7820*	*0.1264*	*0.1948*	*0.8258*
**CDAA diet + Iso CON**	Mean	0.006011	0.102100	0.006681	0.004063
	SEM	0.0003767	0.0033130	0.0003398	0.0001885
P vs. CON diet + Iso CON		*0.6114*	***<0.0001***	*0.7686*	*0.6842*
**CDAA diet + Anti-IL-1ß**	Mean	0.006332	0.099520	0.007934	0.00663
	SEM	0.000238	0.0045640	0.0005314	0.0007758
P vs. CON diet + Anti-IL-1ß mAb		*0.6588*	***<0.0001***	*0.3669*	***0.0036***
P vs. CDAA diet + Iso CON		*0.4764*	*0.7232*	***0.0352***	***0.0059***

Group ID		Normalized to tibia lenght: Heart weight/tibia length (mg/mm)	Liver weight/tibia length (mg/mm)	Kidney weight/tibia length (mg/mm)	Spleen weight/body weight (mg/mg)
**CON diet + Iso CON**	Mean	11.21	78.48	11.65	6.52
	SEM	0.6245	7.4870	0.7072	0.8895
**CON diet + Anti-IL-1ß**	Mean	10.59	101.6	12.76	6.663
	SEM	0.5899	19.6200	0.6304	1.132
P vs. CON diet + Iso CON		*0.4220*	*0.2740*	*0.4056*	*0.9258*
**CDAA diet + Iso CON**	Mean	10.93	188.2	12.21	8.563
	SEM	0.4200	8.9680	0.4665	0.8787
P vs. CON diet + Iso CON		*0.7071*	***<0.0001***	*0.657*	*0.1935*
**CDAA diet + Anti-IL-1ß**	Mean	11.64	185.4	14.73	12.09
	SEM	0.4143	13.9000	1.189	1.191
P vs. CON diet + Anti-IL-1ß mAb		*0.1387*	***0.0001***	*0.1082*	***0.0010***
P vs. CDAA diet + Iso CON		*0.2934*	*0.8747*	***0.0313***	***0.0196***

Supplementary table 2. - Organ weight

Heart, liver, kidney, spleen and tibia was collected and measured. Organ weights were normalized to both body weights and to tibia length. Data is presented as mean ± standard error of mean (SEM). Statistical analysis: two-way ANOVA, Fischer’s LSD post hoc test. Statistically significant is considered $P < 0.05$. Statistically significant P values are highlighted with bold font.
