# Supplementary material for: IL-1β neutralization prevents diastolic dysfunction development, but lacks hepatoprotective effect in an aged mouse model of NASH
Source: Sci Rep. 2023 Jan 7;13:356. doi: 10.1038/s41598-022-26896-3 (PMC9825403; doi:10.1038/s41598-022-26896-3)
Supplement: Supplementary file 3 — Supplementary Table 3. [file 41598_2022_26896_MOESM3_ESM.pdf]

IL-1β neutralization prevents diastolic dysfunction development, but lacks hepatoprotective effect in an aged mouse model of NASH

Dániel Kucsera, PharmD[1,2,3], Viktória E. Tóth, PharmD, PhD[1,2,3], Nabil V. Sayour, MD[1,2,3], Tamás Kovács, MSc [1,2,3], Tamás Gergely, MD [1,2,3], Mihály Ruppert, MD, PhD[4], Tamás Radovits, MD, PhD[4], Alexandra Fábián, MD[4], Attila Kovács, MD, PhD [4], Béla Merkely, MD, PhD[4], Péter Ferdinandy, MD, PhD[1,5], Zoltán V. Varga, MD, PhD[1,2,3]

[1]Department of Pharmacology and Pharmacotherapy, Semmelweis University, Budapest, Hungary;
[2]HCEMM-SE Cardiometabolic Immunology Research Group, Semmelweis University, Budapest, Hungary;
[3]MTA-SE Momentum Cardio-Oncology and Cardioimmunology Research Group, Semmelweis University, Budapest, Hungary;
[4]Heart and Vascular Center, Semmelweis University, Budapest, Hungary;
[5]Pharmahungary Group, Szeged, Hungary.

Corresponding author: Zoltán V. Varga (varga.zoltan@med.semmelweis-univ.hu)

Gene name	Accession number	Forward primer	Reverse primer	Product size (bp)
Il1b	NM_008361.4	GCACTACAGGCTCCGAGATGAAC	TTGTCGTTGCTTGGTTCTCCTTGT	147
Pdcd1 (PD-1)	NM_008798.2	CAAGGACGACACTCTGAAGGAG	TCTTCTCTCGTCCCTGGAAGT	89
Ctla4	NM_009843.4	CCATGCCCGGATTCTGACTT	GGACTTCTTTTCTTTAGCATCTTGC	119
CD274 (PD-L1)	NM_021893.3	CAGCAACTTCAGGGGGAGAG	TTTTGCGGTATGGGGCATTG	176
Myc	NM_010849.4	GTCTTTCCCTACCCGCTCAA	TCTTCTTGCTCTTCTTCAGAGTCG	191
Gpc3	NM_016697.3	CCAGATCATTGACAAACTGAAGCA	CGCAGTCTCCACTTTCAAGTCC	116
Mki67	NM_001081117.2	GACAGCTTCCAAAGCTCACC	TGTGTCCTTAGCTGCCTCCT	230
Afp	NM_007423.4	TGGTTACACGAGGAAAGCCC	AATGTCGGCCATTCCCTCAC	139
Pcna	NM_011045.2	AGATGTGCCCCTTGTTGTAGAG	TGGCATCTCAGGAGCAATCTT	148
Tnfa	NM_013693.3	TACTGAACTTCGGGGTGATTGGTCC	CAGCCTTGTCCCTTGAAGAGAACC	295
Ifng	NM_008337.4	CGGCACAGTCATTGAAAGCC	TGCATCCTTTTTCGCCTTGC	268
Tgfb	NM_021578.2	GACCGCAACAACGCAATCTA	ACCAAGGTAACGCCAGGAAT	207
Ccl2	NM_011333.3	ACCTGCTGCTACTCATTCACC	CTCTTGAGCTTGGTGACAAAAACTA	119
CD80	NM_001359898.1	TTTAGCATCTGCCGGGTGGA	CCCCGGTCTGAAAGGACCAG	488
CD163	NM_001170395.1	GCCTCTGCTGTCACTAACGC	AGATCCATCTGAGCAGGTCACT	354
CD169 (SIGLEC1)	NM_011426.3	ACTATCCAGGAGGAAGTGGTTGC	ACCAGGCCAGGAGGGAAAAC	433
Nos2	NM_010927.4	AGGGACTGAGCTGTTAGAGACA	GTCATCTTGTATTGTTGGGCTGAG	259
Ccn2 (Ctgf)	NM_010217.2	AGCGGTGAGTCCTTCCAAAG	TTCCAGTCGGTAGGCAGCTA	222
Col1a1	NM_007742.4	TCTCCACTCTTCTAGTTCCT	TTGGGTCATTTCCACATGC	226
Col3a1	NM_009930.2	GCTCGAGGCAATGATGGT	ACCCTGCAGGTCCAACTTC	118
Ppia	NM_008907.2	TATCTGCACTGCCAAGACTGAGTG	CTTCTTGCTGGTCTTGCCATTCC	127

Supplementary table 3. - Primer list

Primer sequences (5'→3'), accession number, and product size are shown for the genes used in qRT-PCR analyses.
